# Structural Basis for DNA Recognition by the Two-Component Response Regulator RcsB

**DOI:** 10.1128/mBio.01993-17

**Published:** 2018-02-27

**Authors:** Ekaterina V. Filippova, Bozena Zemaitaitis, Theint Aung, Alan J. Wolfe, Wayne F. Anderson

**Affiliations:** a Center for Structural Genomics of Infectious Diseases, Northwestern University Feinberg School of Medicine, Chicago, Illinois, USA; b Department of Biochemistry and Molecular Genetics, Northwestern University Feinberg School of Medicine, Chicago, Illinois, USA; c Department of Microbiology and Immunology, Loyola University Chicago, Health Sciences Division, Stritch School of Medicine, Maywood, Illinois, USA; d Keck Biophysics Facility, Northwestern University, Evanston, Illinois, USA; Columbia University

**Keywords:** DNA-binding proteins, transcription, two-component signal transduction, X-ray crystallography

## Abstract

RcsB is a highly conserved transcription regulator of the Rcs phosphorelay system, a complex two-component signal transduction system (N. Majdalani and S. Gottesman, Annu Rev Microbiol 59:379–405, 2005; A. J. Wolfe, Curr Opin Microbiol 13:204–209, 2010, https://doi.org/10.1016/j.mib.2010.01.002; D. J. Clarke, Future Microbiol 5:1173–1184, 2010, https://doi.org/10.2217/fmb.10.83). RcsB plays an important role in virulence and pathogenicity in human hosts by regulating biofilm formation. RcsB can regulate transcription alone or together with its auxiliary transcription regulators by forming heterodimers. This complexity allows RcsB to regulate transcription of more than 600 bacterial genes in response to different stresses (D. Wang et al., Mol Plant Microbe Interact 25:6–17, 2012, https://doi.org/10.1094/MPMI-08-11-0207). Despite increasing knowledge of RcsB importance, molecular mechanisms that drive the ability of RcsB to control transcription of a large number of genes remain unclear. Here, we present crystal structures of unphosphorylated RcsB in complex with the consensus DNA-binding sequence of 22-mer (DNA22) and 18-mer (DNA18) of the *flhDC* operon from *Escherichia coli* determined at 3.15- and 3.37-Å resolution, respectively. The results of our structural analysis combined with the results of *in vitro* binding assays provide valuable insights to the protein regulatory mechanism, demonstrate how RcsB recognizes target DNA sequences, and reveal a unique oligomeric state that allows RcsB to form homo- and heterodimers. This information will help us understand the complex mechanisms of transcriptional regulation by RcsB in bacteria.

## IMPORTANCE

RcsB is a well-studied two-component response regulator of the Rcs phosphorelay system, conserved within the family *Enterobacteriaceae*, which includes many pathogens. It is a global regulator, controlling more than 5% of bacterial genes associated with capsule biosynthesis, flagellar biogenesis, cell wall biosynthesis, antibiotic resistance, biofilm formation, and virulence in pathogens. Knowledge of RcsB structure represents a unique opportunity to explore mechanisms that regulate the Rcs phosphorelay system and its role in the family *Enterobacteriaceae*.

## OBSERVATION

Two-component signal transduction (TCST) systems play important roles in regulating many bacterial processes, primarily in response to altered cellular environments (1). The Rcs phosphorelay system is one of the most studied bacterial TCST systems; it is also one of the most complex (see Fig. S1A in the supplemental material). At its core are three multidomain proteins, two hybrid histidine kinases (RcsC and RcsD) and a response regulator (RcsB). Phosphotransfer within the Rcs phosphorelay system is induced mainly by damage to the cell envelope (2). Phosphorylation of RcsB is thought to induce a functionally active form that facilitates its ability to regulate gene expression (3). The complexity of this phosphorelay is extended by RcsB’s ability to associate with other transcription regulators and form protein complexes, in some of which RcsB is phosphorylated and in some it is not (4–9). For example, phosphorylated RcsB can partner with an unstable RcsA transcription regulator to bind to a DNA sequence (termed the RcsAB box) located in the vicinity of promoters for several genes, including the *cps* operon, which encodes components involved in capsular polysaccharide biosynthesis (4). A similar sequence is located near the promoter for the *flhDC* operon, which encodes the master regulator of flagellar biogenesis (5, 10). In its phosphorylation-independent state, RcsB can interact with various transcription regulators, including GadE, BglJ, RmpA, PhoP, MatA, and RflM (6–9). This complexity allows RcsB to regulate diverse bacterial characteristics and cellular processes, many of which affect pathogenesis in humans (3, 11, 12).

10.1128/mBio.01993-17.2 FIG S1  Schematic representation of the Rcs phosphorelay system in *E. coli*. (A) The Rcs phosphorelay system contains three core components,: RcsC, RcsD, and RcsB. Phosphotransfer within the Rcs phosphorelay system occurs through two additional proteins, RcsF, an outer membrane (OM) lipoprotein, and IgaA, an inner membrane (IM) uncharacterized protein. In response to signals received from RcsF and Iga, the kinase RcsC initiates a phosphorelay cascade by transferring a phosphoryl group from ATP to a conserved histidinyl residue within its HKA (histidine kinase A phospho-acceptor) domain and then to a conserved aspartyl residue in its REC (receiver) domain. The phosphoryl group then passes to a histidinyl residue in the HPT (histidine phosphotransferase) domain of RcsD, and finally to a conserved aspartyl residue in the REC domain of RcsB. In the absence of such signals, the RcsC-RcsD complex possesses net phosphatase activity that removes phosphoryl groups from phospho-RcsB. When carbon flux exceeds the capacity of central metabolic pathways, the central metabolite acetyl-phosphate (acP) can donate its phosphoryl group to RcsB in a nonenzymatic process. RcsB partners with other transcription factors (TF), such as RcsA with HTH DNA-binding and regulatory domain (RD) to form heterocomplexes. Additional protein domain abbreviations: SD, signal domain; HK, histidine kinase ATP-binding domain; ABL, alpha-beta-loop domain. (B) Functional domains of RcsB. Download FIG S1, TIF file, 2.59 MB. Copyright © 2018 Filippova et al. 2018 Filippova et al. This content is distributed under the terms of the Creative Commons Attribution 4.0 International license.

The lack of a crystal structure has been a major obstacle in understanding (i) the molecular mechanisms by which RcsB activates transcription from a wide range of promoters, (ii) the formation of phosphorylation-dependent and phosphorylation-independent protein complexes, and (iii) the mechanisms of RcsB dimerization and DNA binding. RcsB belongs to the FixJ/NarL family of response regulators with two domains connected by a long linker (Fig. S1B). The C-terminal DNA-binding domain (helix-turn-helix [HTH]) of RcsB has a four-helix bundle fold with a classical helix-turn-helix DNA recognition motif (13, 14). The N-terminal REC (receiver) domain of RcsB is composed of a five-stranded central β-sheet surrounded by five α-helices and contains the conserved aspartyl residue (D56) that serves as the phosphoryl acceptor (15). *In vivo* analysis shows that D56 phosphorylation is essential for activation of transcription from those promoters that require the RcsB homodimer or the RcsB-RcsA heterodimer (10, 16). Previously, the structures of full-length RcsB or its complex with DNA site had not been obtained. Instead, structures of the isolated RcsB domains had been determined (14, 15).

### *In vitro* binding analysis of RcsB-DNA interactions.

**FIG 1:**
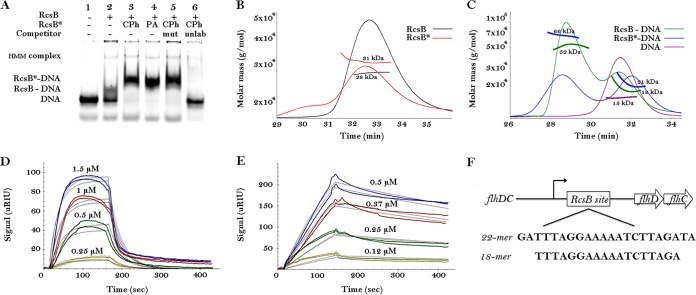
Oligomeric state and DNA binding activity of RcsB. (A) EMSA of RcsB against DNA22. Lane 1, free DNA; lane 2, DNA in the presence of RcsB; lane 3, DNA in the presence of RcsB and carbamoyl phosphorylate (CPh); lane 4, DNA in the presence of RcsB and phosphoramide (PA); lane 5, DNA in the presence of RcsB, CPh, and unlabeled mutated (mut) DNA22; lane 6, DNA in the presence of RcsB, CPh, and unlabeled (unlab) wild-type DNA22. HMM complex, higher-molecular-mass complex. (B) The SEC-MALS elution profiles of RcsB and RcsB in the presence of phosphodonor (RcsB*). The horizontal bold lines show the determined molecular masses (MM). The theoretical MM of the RcsB monomer is 24 kDa. (C) The SEC-MALS elution profiles of RcsB and RcsB* (with phosphodonor) in the presence of DNA22. The horizontal bold lines show the calculated MM of the RcsB-DNA complex. The theoretical MM of the RcsB dimer bound to the 22-bp DNA duplex is 63 kDa. The second peak eluted around 31 min corresponds to unbound DNA. (D and E) SPR sensograms (colored curves) of unphosphorylated and carbamoyl-phosphorylated RcsB and DNA22, respectively. *K*_*D*_ values were calculated based on a 1:1 kinetic model, in which one RcsB dimer interacts with one double-stranded DNA. The fitting curves are shown in black. The corresponding concentrations of RcsB are indicated above the SPR curves. (F) RcsB-binding site from the *flhDC* promoter and DNA22 and DNA18 sequences.

To understand RcsB function and particularly its mechanisms for DNA recognition, we first determined requirements for RcsB-DNA complex formation *in vitro* and then determined its cocrystal structures. We performed gel mobility assays (electrophoretic mobility shift assay [EMSA]), which confirmed that RcsB specifically binds the RcsAB box from the *flhDC* promoter, in the presence or absence of phosphodonors, either carbamoyl phosphate (CPh) or phosphoramidate (PA) (Fig. 1A). RcsB bound DNA in the absence of a phosphoryl donor, but the complex appeared to have reduced stability. The presence of phosphoryl donors appeared to increase binding affinity, as determined by titration EMSA with different RcsB concentrations (Fig. S2). To test whether RcsB forms a dimer in solution or upon binding to DNA and to determine whether phosphorylation affects dimer formation, we performed size exclusion chromatography with multiangle light scattering (SEC-MALS) analysis. In the absence of DNA, RcsB existed predominantly as a monomer in solution even if the phosphoryl donor CPh was present (Fig. 1B). Thus, in the absence of DNA, either CPh does not phosphorylate the RcsB monomer, or phosphorylation does not favor RcsB dimerization. In the presence of DNA, however, RcsB bound DNA and formed a dimer with or without CPh (Fig. 1C). SEC-MALS analysis also confirmed that the unphosphorylated RcsB-DNA complex is less stable and prone to dissociation (Fig. 1C). Based on the increased apparent molecular mass (MM) of the complex, our data suggest that the presence of DNA enhances RcsB phosphorylation and favors dimerization. Surface plasmon resonance (SPR) analysis provided evidence that phosphorylation increases binding affinity of RcsB for its DNA site (Fig. 1D and E). The RcsB in the presence of a phosphodonor binds more tightly (equilibrium dissociation constant [*K*_*D*_] of ~0.16 μM) than in the absence of CPh (*K*_*D*_ of ~5.34 μM).

10.1128/mBio.01993-17.3 FIG S2  EMSA titration of the 22-bp DNA fragment with *E. coli* RcsB protein. Lane 1, free DNA; lane 2, DNA in the presence of RcsB and phosphoramide (PA); lanes 3 to 10, DNA in the presence of RcsB at different concentrations and PA at a concentration of 20 mM. The RcsB concentrations in lanes 1 to 10 were 0, 104, 31, 42, 52, 62, 73, 83, 104, and 125 nM, respectively. Download FIG S2, TIF file, 0.29 MB. Copyright © 2018 Filippova et al. 2018 Filippova et al. This content is distributed under the terms of the Creative Commons Attribution 4.0 International license.

### Structure of the RcsB-DNA complex.

**FIG 2:**
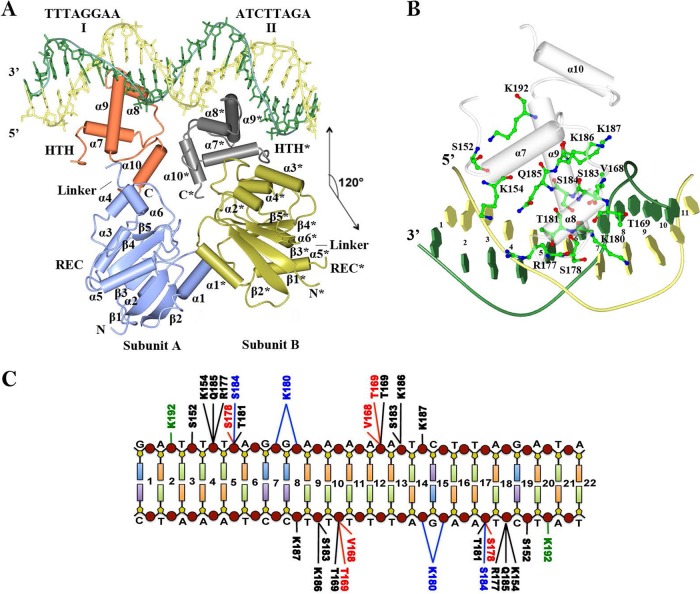
Structure of the RcsB-DNA complex. (A) Structure of the RcsB homodimer bound to DNA22. The rotational two-fold axis of symmetry in the dimer of REC and HTH domain are shown as coordinate axes *x* and *z* at a 120°. The secondary structure elements are labeled (marked with an asterisk for subunit B). The main and complementary strands of DNA are shown in yellow and green, respectively. (B) RcsB HTH domain (worms/tubes) bound to half-site I of DNA22 (worms/blocks). The residues involved in interactions are shown as balls and sticks. (C) Summary of interactions between RcsB residues and DNA nucleotides (indicated by lines). Hydrogen bonds (H-bonds) between protein side chains and DNA bases are shown in blue, H-bonds between main chains and phosphates of DNA are shown in red, and H-bonds between side chains and phosphates of DNA are shown in black. The electrostatic interactions (<3.8 Å) between RcsB and DNA are shown in green.

To understand the structural basis for DNA recognition by RcsB, we crystallized unphosphorylated RcsB in the presence of DNA that contains the RcsB binding site from the *flhDC* promoter region of *Escherichia coli* (Fig. 1F). This binding site can bind both the RcsB homodimer and the RcsA-RcsB heterodimer (5, 17, 18). We determined two crystal structures of full-length RcsB bound to 22-mer (DNA22) or 18-mer (DNA18) of the *flhDC* operon from *E*. *coli* (Fig. 2A and Fig. S3). For details of crystallization, data collection, and structure determination, see Text S1 and Table S1 in the supplemental material. The asymmetric unit of the RcsB crystal in complex with DNA22 contained one RcsB dimer bound to one double helix DNA (Fig. 2A), whereas the RcsB crystal in complex with DNA18 contained an RcsB tetramer (two RcsB dimers) bound to two parallel double helical DNAs (Fig. S3). In both crystal forms, the RcsB dimers displayed similar structures, and the DNA had a classical B form with an average base pair rise and twist of 3.2 Å and 34°, respectively; the DNA was slightly bent along the surfaces of the RcsB HTH domains. The structural similarity between RcsB dimers bound to DNA observed in two different crystal forms suggests that this dimer is likely biologically relevant.

10.1128/mBio.01993-17.1 TEXT S1  Supplemental Materials and Methods. Download TEXT S1, DOCX file, 0.09 MB. Copyright © 2018 Filippova et al. 2018 Filippova et al. This content is distributed under the terms of the Creative Commons Attribution 4.0 International license.

10.1128/mBio.01993-17.4 FIG S3  Structure of the RcsB tetramer bound to two DNAs. Structure of the RcsB tetramer bound to two DNAs (18-mer): front (A), side (B), and top (C) views. In the structure, the arrangement of the domains of the dimers of RcsB was similar to that of the RcsB dimer found in complex with DNA22. Superposition of dimers in the two structures shows that the average RMSD value was 1.15 Å. The secondary structure elements of subunit A are labeled on panel A. The main and complementary strands of DNAs are colored in yellow and red and in green and blue, respectively. The secondary structure elements involved in intersubunit interactions between dimers of the RcsB tetramer are shown on panel B. Download FIG S3, TIF file, 1.79 MB. Copyright © 2018 Filippova et al. 2018 Filippova et al. This content is distributed under the terms of the Creative Commons Attribution 4.0 International license.

10.1128/mBio.01993-17.6 TABLE S1  Crystal cell parameters, X-ray data collection, and structure refinement statistics. Download TABLE S1, DOCX file, 0.05 MB. Copyright © 2018 Filippova et al. 2018 Filippova et al. This content is distributed under the terms of the Creative Commons Attribution 4.0 International license.

### Structure of the RcsB homodimer bound to DNA.

The RcsB dimer observed in two different crystal forms is asymmetric (Fig. 2A and Fig. S3). Subunit A (or C) was bound to the first DNA half-site, whereas subunit B (or D) was bound to the second half-site. The subunits in the dimer were not related by a two-fold rotation axis of symmetry. Instead, an approximate two-fold axis was present between each of the RcsB domains of a dimer. However, these two axes met each other at an angle of about 120°. In the RcsB dimer, the domains superimpose with an average root mean square deviation (RMSD) value of 0.9 Å (for 129 C_α_ atoms) and 0.6 Å (for 57 C_α_ atoms) for the REC and HTH domains, respectively. The main differences were found in the position of the partially disordered linker region between helices α5 of the REC domain and α7 of the HTH domain. The structure of the RcsB asymmetric dimer is unique because it does not correspond to any known structure of full-length TCST response regulators despite the fact that dimer asymmetry of some TCST response regulators in their DNA-bound complexes has been reported previously. Examples include structures of the quorum-sensing transcriptional factor TraR from the LuxR family in the presence of its ligand, the pheromone *N*-3-oxooctanoyl-l-homoserine lactone (OOHL) (19), and of the response regulators PhoP and KdpE from the OmpR/PhoP family determined in their unphosphorylated state (20, 21). In each case, the asymmetry defines the regulator’s distinct dimeric structure, its interactions with DNA, and thus, the response regulator’s individual functions. The ability of two-component response regulators, including RcsB, PhoP, and KdpE, to bind to DNA in their unphosphorylated state might not be a coincidence and could be important for these proteins’ regulatory activities.

### RcsB-DNA contacts.

In the RcsB-DNA complex, the helices that form the HTH DNA recognition motif of the DNA-binding domain (α8 and α9) bound to the floor of the DNA major groove perpendicular to the DNA helical axis (Fig. 2A and Fig. S3A). The RcsB-DNA interactions were largely identical at all sites in the structure of RcsB in complex with either DNA22 or DNA18 (Fig. 2B). In these structures, two amino acid residues of RcsB made direct sequence-specific contacts with only three bases of the DNA. K180 and S184, located on helix α9, made hydrogen bonds (H-bonds) with G_-8_, A_−9_ and A_−6_ in one-half-site (TTAGGAA) or with G_−30_, A_−31_, and A_−28_ in its complement (TCTAAGA) on the other half-site of the DNA (Fig. 2C). Most H-bonds occurred between phosphates of the DNA backbone and side chains of several residues of the RcsB HTH domain (S152, K154, T169, R177, T181, S183, Q185, K186, and K187). Some main chain atoms from V168, T169, and S178 contacted phosphates of the DNA backbone. The side chain amino group of K192 in the RcsB-DNA22 complex contributes to electrostatic interactions, but it is too far away for a direct H-bond (Fig. 2C). The resolution of the crystal structures did not allow detection of any water-mediated interactions. Biochemical and physiological experiments have shown that K180 is a critical residue for RcsB-dependent regulation of *flhDC* (22). Nε-lysine acetylation, which can negate the positive charges of K154 and K180, is reported to reduce RcsB’s ability to bind the *flhDC* DNA site (22, 23). Our structures support the importance of both and suggest that the small number of DNA sequence-specific contacts with DNA bases likely permits RcsB to function on multiple promoters.

### Intersubunit domain and dimer interfaces.

In the asymmetric dimer formed upon binding to DNA (Fig. 2A; Fig. S4A and Fig. S4B), both RcsB domains contributed to the dimer interface, and the subunits in the dimer were stabilized primarily through hydrophobic contacts. In contrast, the linker region between the REC and HTH domains ran along the side of each subunit in both structures and was not involved in dimerization. Very few H-bonds and no salt bridges or water molecules were found in the dimerization interface. Interestingly, within the RcsB tetramer of the RcsB-DNA18 complex, dimers formed asymmetric interfaces between their REC domains (Fig. S3B and Fig. S4B). We cannot exclude the existence of a higher-MM RcsB-DNA complex, as indicated by the slowest-migrating band (Fig. 1A). This putative complex could correspond to an RcsB tetramer bound to two DNAs as seen in our RcsB-DNA18 cocrystal structure. Further analysis is needed to confirm this supposition. In all crystallized dimers, the HTH domains interacted through helix α10 and the loop between helices α7 and α8 (Fig. 2A and Fig. S4A). The orientation and binding mode of RcsB HTH domains resemble those seen in the structure of the isolated DNA-binding domain of the NarL regulator in complex with DNA (24). The REC domains in the RcsB dimer interacted through helix α1 and the loops between β1 and α1 and between β5 and α5 (Fig. 2A and Fig. S4B). Previously, we reported that isolated RcsB REC domains could form a similar dimer in the crystal and characterized the dimerization interface (15).

10.1128/mBio.01993-17.5 FIG S4  Interdomain and intersubunit interfaces in RcsB asymmetric dimer. (A) Structure of the HTH domains (worms/tubes) of the RcsB dimer bound to DNA22 and DNA18 (surface model). (B, left) Structure of the REC domains (worms/tubes) of the RcsB dimer in the cocrystal structure with DNA22. (Right) Structure of RcsB REC domains in the cocrystal structure with DNA18. Secondary structure elements involved in intersubunit interactions are labeled. The larger buried surface area between HTH and REC domains (indicated below) in the RcsB dimer bound to DNA22 likely contributes to more stable complex, as SEC-MALS and SPR did not observe complex formation and binding between the RcsB and DNA18 (data not shown). (C and D) Ribbon diagram of subunit A (C) and subunit B (D) of the asymmetric RcsB dimer. Secondary structure elements involved in interdomain interface are labeled. Download FIG S4, TIF file, 2.69 MB. Copyright © 2018 Filippova et al. 2018 Filippova et al. This content is distributed under the terms of the Creative Commons Attribution 4.0 International license.

The subunits of the RcsB dimer had different conformations and interdomain interfaces that involve a distinct set of contacts (Fig. 2A and Fig. S4C). The area of surface contacts between the HTH and REC domain were two times larger in subunit B (D) than in subunit A (C). In subunit A (C), helices α4 and α6 of the REC domain interacted with helices α9 and α10 of the HTH domain. In contrast, in subunit B (D), helix α4 and the β4-α3 loop of the REC domain interacted with helices α7 and α8 of the HTH domain. The unusual asymmetric oligomeric state(s) of RcsB could present different surfaces for interactions with RNA polymerase (RNAP) and thus extend RcsB’s capacity to regulate genes with a variety of promoter architectures. At the same time, this unique asymmetry and the presence of hydrophobic interactions between RcsB subunits may allow unphosphorylated RcsB to form heterodimers with auxiliary transcription regulators (4–9). For example, the transcription regulator RflM in *Salmonella enterica* serovar Typhimurium increases the binding affinity of unphosphorylated RcsB for the homologous RcsB DNA-binding site located on the *flhDC* operon (9) and therefore can form an analogous asymmetric dimer. Undoubtedly, our structures will provide important details for investigating interactions between RcsB and auxiliary transcription factors.

Only a small number of full-length FixJ/NarL response regulators, including NarL, VraR, and ChrA, have been structurally characterized (24–27); however, the structure of a full-length response regulator from the FixJ/NarL family in its DNA-bound state has not been determined previously. Structural comparisons of full-length FixJ/NarL regulators using the Dali server (28) show that the RcsB asymmetric dimer and/or RcsB subunits do not correspond to any existing structure despite the fact that the structural fold and dimerization interfaces of the isolated RcsB HTH and REC domains are consistent with structures of homologous transcription factors (15, 24–27). The relative orientation of isolated domains, conformation of the interdomain linker, and oligomerization of subunits in the determined RcsB structures are different compared to structures of homologous proteins. These differences likely contribute to RcsB’s function.

### Alternative mechanism of transcriptional regulation by RcsB.

One of the features of our crystal structures is that RcsB forms an asymmetric dimer that is identical in two different crystal forms. In one structure, unphosphorylated RcsB exists as a dimer bound to one double-stranded DNA; in another, two RcsB dimers form a tetramer bound to two parallel double-stranded DNAs. These structures combined with *in vitro* binding assays reveal that unphosphorylated RcsB can form a dimer upon binding to DNA and emphasize that the presence of a phosphoryl donor in the presence of DNA favors dimerization and enhances RcsB binding affinity. It is widely accepted that phosphorylation facilitates formation of the RcsB dimer, which can then bind its DNA site and thus regulate transcription. Under environmental conditions that favor phosphorylation of RcsB either by its cognates RcsC/RcsD or by the small molecule phosphoryl donor acetylphosphate, formation of the phosphorylated dimer could be favored prior to binding its DNA site. However, because RcsB in its unphosphorylated state can bind to DNA and can activate expression of some genes (29), we hypothesize that the RcsB unphosphorylated state is important for function and that an alternate mechanism may also exist. In this alternative, unphosphorylated RcsB binds DNA as observed in the structures, albeit as an unstable RcsB-DNA complex that can be stabilized by phosphorylation of RcsB or through interactions with its cognates RcsC and/or RcsD, as observed for the OmpR regulator (30). We further hypothesize that phosphorylation would induce conformation changes in the receiver domain, propagating structural rearrangements within intersubunit interfaces of the RcsB homodimer that subsequently lead to stabilization of RcsB-DNA interactions. Since the unphosphorylated complex is less stable, reversible phosphorylation may provide an extra opportunity to regulate recruitment and stabilization of RNA polymerase and thus transcription. In this case, phosphorylation of RcsB could regulate the level of gene expression. In its lower-affinity unphosphorylated state, RcsB could also easily dissociate from DNA or form heterodimers with other transcription regulators. This mechanism combined with asymmetry of the RcsB dimer and low specificity to DNA-binding sites may explain RcsB’s ability to regulate a diverse set of genes. At the same time, we anticipate that phosphorylation may enhance formation of a different RcsB dimer or another RcsB oligomer prior to or upon binding to DNA. Therefore, future experiments are needed to explore these possibilities.

### Conclusions.

Here, we described the crystal structures of unphosphorylated full-length RcsB from *E. coli* in complex with one of its DNA sites. Our structural data support the hypothesis that an RcsB dimer forms upon binding to DNA. The data emphasize that phosphorylation enhances RcsB binding affinity and presumably facilitates interaction with RNAP and thus activates transcription. The structure yields the first view of the RcsB homodimer in its unphosphorylated state bound to the DNA and provides an explanation for how RcsB could regulate large numbers of genes. The RcsB-DNA complex structures provide insights into the structural basis of the mechanism(s) of transcriptional regulation by RcsB and offer a platform for design of novel antimicrobial compounds against Gram-negative pathogens.

### Methods. (i) Gel mobility shift assays.

DNA strands of 22 or 18 bp (as defined in Text S1 in the supplemental material) 5′ end labeled with IRDye 700 or not labeled were annealed according to the general methodology for infrared electrophoretic mobility shift assay (LI-COR Biosciences). DNA (4.15 nM) was then incubated in 1× binding buffer (10 mM Tris, 40 mM KCl, 1 mM dithiothreitol [DTT] [pH 7.5], 10 mM MgCl_2_, 5% glycerol, and 0.1 μg/μl of bovine serum albumin) in the presence or absence of 169 nM purified RcsB in a total volume of 20 μl for 20 min at room temperature. The samples (10 μl per well) were loaded onto a 7% native acrylamide/bisacrylamide (29:1) 1× Tris-glycine-EDTA (TGE) gel and ran in 1× TGE buffer for 40 min in a cold room. After electrophoresis, the gel was scanned on an Odyssey imager (LI-COR Biosciences Inc.) For competition reactions, 100-fold excess of unlabeled DNA22 or DNA18 or mutated DNA22 or DNA18 (as defined in Text S1) in the binding buffer (described above in a total volume of 20 μl) was added along with labeled DNA (415 nM). For phosphorylation, phosphoramidate (PA) or carbamoyl phosphate (CPh) was added to a final concentration of 20 mM to the binding reaction mixture and incubated for 30 min at 37°C in a heating block prior to the addition of the DNA. For titration experiments, increasing concentrations of RcsB in the binding reaction mixtures were used (as given in the legend to Fig. S2), and 10 μl of each reaction mixture was loaded onto a 8% native gel. The conditions of the binding reaction and electrophoresis were kept the same. All assays were repeated four times to confirm reproducibility.

### (ii) Size exclusion chromatography with multiangle light scattering.

The molecular weights of RcsB (with and without CPh), as well as their respective complexes with DNA22 and DNA18 fragments used in cocrystallization experiments, were determined by conducting size exclusion chromatography with multiangle light scattering (SEC-MALS) experiments using Agilent 1260 high-performance liquid chromatography (HPLC) system (Agilent Technologies) equipped with a Dawn HeleosII 18-angle MALS detector, Optilab T-rEX (*r*efractometer with *ex*tended range) refractive index detector, WyattQELS quasielastic (dynamic) light scattering (QELS) detector, and ASTRA software (all four from Wyatt Technology Europe GmbH). All runs with DNA and/or unphosphorylated RcsB were conducted in 10 mM Tris-HCl buffer at pH 8.3 with 150 mM NaCl and 0.5 mM Tris(2-carboxyethyl)phosphine (TCEP) on a Superdex 75 10/300 GL column (GE Healthcare) preequilibrated with the same buffer at a flow rate of 0.5 ml/min at 22°C. Runs with phosphorylated RcsB were conducted in 10 mM Tris-HCl buffer at pH 8.3 with 150 mM NaCl, 10 mM MgCl_2_, and 0.5 mM TCEP. To phosphorylate RcsB, 50 mM CPh and 10 mM MgCl_2_ were added to the protein solution prior to experiments. A total of 270 µl of protein solution containing RcsB (both in the presence and absence of phosphodonor) and respective RcsB complexes with DNA22 and DNA18 was injected onto the column. The final concentration of RcsB and DNA22 or DNA18 used in experiments was 1 mg/ml. A refractive index increment (*dn*/*dc*) value of 0.1600 was used for DNA samples, and a value of 0.178 was used for proteins and their complexes with DNA. Lysozyme from chicken egg white (Sigma-Aldrich Corp.) was used as a control.

### (iii) Surface plasmon resonance.

Binding of RcsB (with and without CPh) to DNA22 and DNA18 fragments used in cocrystallization trials was measured using a four-channel Reichert surface plasmon resonance (SPR) instrument (Reichert Technologies). Biotinylated DNA22 or DNA18 (0.5 mg/ml) was captured onto NeutrAvidin planar mSAM surface (catalog no. 13206065; Reichert Technologies, Depew, NY) using high-affinity binding of biotin to avidin (at a flow rate of 33 µl min^−1^ for 3 min) until a signal increase of approximately 600 micro-refractive index units (μRIUs) was achieved. The biotinylated DNA fragments were synthesized by IDT Inc. The protein, DNA, and running buffer solutions used in all experiments contained 10 mM Tris-HCl (pH 8.3) with 150 mM NaCl and 0.5 mM TCEP. Phosphorylation of RcsB was achieved by adding 50 mM CPh and 10 mM MgCl_2_ into the protein solution. All SPR experiments were performed at 25°C. RcsB (0.25, 0.5, 1, and 1.5 µM) and RcsB phosphorylated with carbamoyl phosphate (0.125, 0.25, 0.375, and 0.5 µM) were injected over the chip at a flow rate of 30 µl min^−1^ for 2.5 min. Dissociation was observed for 5 min, and regeneration of the chip was carried out by a 1.5-min injection of 1 M Tris-HCl (pH 4.0) at 40 µl min^−1^. Binding was detected as a change in the refractive index at the surface of the chip, as measured by response units (µRIUs). A reference flow channel was used to record the background response, and background was subtracted from each sample injection. Equilibrium dissociation constant (*K*_*D*_) values were calculated as ratios of association rate (*k*_*a*_)/dissociation rate (*k*_*d*_) determined from kinetic experiments. Each experiment included duplicates of each solute concentration obtained at two channels. Data models were fit using TraceDrawer data analysis software available through Reichert Technologies.

### Data accessibility.

The atomic coordinates and structure factors for RcsB-DNA22 and RcsB-DNA18 have been deposited into the Protein Data Bank (http://www.rcsb.org) with accession numbers or codes 5W43 and 5VXN, respectively. The diffraction images (target identifier [ID] IDP91817) are available at the CSGID website (http://www.csgid.org/csgid/pages/home).

## References

[1] Goudreau PN, Stock AM 1998 Signal transduction in bacteria: molecular mechanisms of stimulus-response coupling. Curr Opin Microbiol 1:160–169. doi:10.1016/S1369-5274(98)80006-4. 10066483

[2] Cho SH, Szewczyk J, Pesavento C, Zietek M, Banzhaf M, Roszczenko P, Asmar A, Laloux G, Hov AK, Leverrier P, Van der Henst C, Vertommen D, Typas A, Collet JF 2014 Detecting envelope stress by monitoring β-barrel assembly. Cell 159:1652–1664. doi:10.1016/j.cell.2014.11.045. 25525882

[3] Clarke DJ 2012 The Rcs phosphorelay: biofilm formation and virulence in the Enterobacteriaceae. Two-component systems in bacteria. Caister Academic Press, Haverhill, United Kingdom.

[4] Brill JA, Quinlan-Walshe C, Gottesman S 1988 Fine-structure mapping and identification of two regulators of capsule synthesis in *Escherichia coli* K-12. J Bacteriol 170:2599–2611. doi:10.1128/jb.170.6.2599-2611.1988. 2836365 PMC211177

[5] Wehland M, Bernhard F 2000 The RcsAB box. Characterization of a new operator essential for the regulation of exopolysaccharide biosynthesis in enteric bacteria. J Biol Chem 275:7013–7020. doi:10.1074/jbc.275.10.7013. 10702265

[6] Castanié-Cornet MP, Cam K, Bastiat B, Cros A, Bordes P, Gutierrez C 2010 Acid stress response in *Escherichia coli*: mechanism of regulation of *gadA* transcription by RcsB and GadE. Nucleic Acids Res 38:3546–3554. doi:10.1093/nar/gkq097. 20189963 PMC2887963

[7] Salscheider SL, Jahn A, Schnetz K 2014 Transcriptional regulation by BglJ-RcsB, a pleiotropic heteromeric activator in *Escherichia coli*. Nucleic Acids Res 42:2999–3008. doi:10.1093/nar/gkt1298. 24335284 PMC3950696

[8] Pannen D, Fabisch M, Gausling L, Schnetz K 2016 Interaction of the RcsB response regulator with auxiliary transcription regulators in *Escherichia coli*. J Biol Chem 291:2357–2370. doi:10.1074/jbc.M115.696815. 26635367 PMC4732218

[9] Kuhne C, Singer HM, Grabisch E, Codutti L, Carlomango T, Scrima A, Erhardt M 2016 RflM mediates target specificity of the RcsCDB phosphorelay system for transcriptional repression of flagellar synthesis in *Salmonella enterica*. Mol Microbiol 101:841–855. doi:10.1111/mmi.13427. 27206164

[10] Francez-Charlot A, Laugel B, Van Gemert A, Dubarry N, Wiorowski F, Castanié-Cornet M-P, Gutierrez C, Cam K 2003 RcsCDB His-Asp phosphorelay system negatively regulates the *flhDC* operon in *Escherichia coli*. Mol Microbiol 49:823–832. doi:10.1046/j.1365-2958.2003.03601.x. 12864862

[11] Latasa C, García B, Echeverz M, Toledo-Arana A, Valle J, Campoy S, García-del Portillo F, Solano C, Lasa I 2012 Salmonella biofilm development depends on the phosphorylation status of RcsB. J Bacteriol 194:3708–3722. doi:10.1128/JB.00361-12. 22582278 PMC3393492

[12] Hinchliffe SJ, Howard SL, Huang YH, Clarke DJ, Wren BW 2008 The importance of the Rcs phosphorelay in the survival and pathogenesis of the enteropathogenic yersiniae. Microbiology 154:1117–1131. doi:10.1099/mic.0.2007/012534-0. 18375804

[13] Takeda Y, Ohlendorf DH, Anderson WF, Matthews BW 1983 DNA-binding proteins. Science 221:1020–1026. doi:10.1126/science.6308768. 6308768

[14] Pristovsek P, Sengupta K, Löhr F, Schäfer B, von Trebra MW, Rüterjans H, Bernhard F 2003 Structural analysis of the DNA-binding domain of the *Erwinia amylovora* RcsB protein and its interaction with the RcsAB box. J Biol Chem 278:17752–17759. doi:10.1074/jbc.M301328200. 12740396

[15] Filippova EV, Wawrzak Z, Ruan J, Pshenychnyi S, Schultz RM, Wolfe AJ, Anderson WF 2016 Crystal structure of nonphosphorylated receiver domain of the stress response regulator RcsB from *Escherichia coli*. Protein Sci 25:2216–2224. doi:10.1002/pro.3050. 27670836 PMC5119572

[16] Majdalani N, Hernandez D, Gottesman S 2002 Regulation and mode of action of the second small RNA activator of RpoS translation, RprA. Mol Microbiol 46:813–826. doi:10.1046/j.1365-2958.2002.03203.x. 12410838

[17] Fredericks CE, Shibata S, Aizawa S, Reimann SA, Wolfe AJ 2006 Acetyl phosphate-sensitive regulation of flagellar biogenesis and capsular biosynthesis depends on the Rcs phosphorelay. Mol Microbiol 61:734–747. doi:10.1111/j.1365-2958.2006.05260.x. 16776655

[18] Wehland M, Kiecker C, Coplin DL, Kelm O, Saenger W, Bernhard F 1999 Identification of an RcsA/RcsB recognition motif in the promoters of exopolysaccharide biosynthetic operons from *Erwinia amylovora* and *Pantoea stewartii* subspecies *stewartii*. J Biol Chem 274:3300–3307. doi:10.1074/jbc.274.6.3300. 9920870

[19] Zhang R-Q, Pappas T, Brace JL, Miller PC, Oulmassov T, Molyneaux JM, Anderson JC, Bashkin JK, Winans SC, Joachimiak A 2002 Structure of a bacterial quorum-sensing transcription factor complexed with pheromone and DNA. Nature 417:971–974. doi:10.1038/nature00833. 12087407

[20] He X, Wang L, Wang S 2016 Structural basis of DNA sequence recognition by the response regulator PhoP in *Mycobacterium tuberculosis*. Sci Rep 6:24442. doi:10.1038/srep24442. 27079268 PMC4832192

[21] Narayanan A, Kumar S, Evrard AN, Paul LN, Yernool DA 2014 An asymmetric heterodomain interface stabilizes a response regulator-DNA complex. Nat Commun 5:3282. doi:10.1038/ncomms4282. 24526190 PMC4399498

[22] Thao S, Chen C-S, Zhu H, Escalante-Semerena JC 2010 N^ε^-lysine acetylation of a bacterial transcription factor inhibits its DNA-binding activity. PLoS One 5:e15123. doi:10.1371/journal.pone.0015123. 21217812 PMC3013089

[23] Hu LI, Chi BK, Kuhn ML, Filippova EV, Walker-Peddakotla AJ, Bäsell K, Becher D, Anderson WF, Antelmann H, Wolfe AJ 2013 Acetylation of the response regulator RcsB controls transcription from a small RNA promoter. J Bacteriol 195:4174–4186. doi:10.1128/JB.00383-13. 23852870 PMC3754749

[24] Maris AE, Sawaya MR, Kaczor-Grzeskowiak M, Jarvis MR, Bearson SMD, Kopka ML, Schröder I, Gunsalus RP, Dickerson RE 2002 Dimerization allows DNA target site recognition by the NarL response regulator. Nat Struct Biol 9:771–778. doi:10.1038/nsb845. 12352954

[25] Leonard PG, Golemi-Kotra D, Stock AM 2013 Phosphorylation-dependent conformational changes and domain rearrangements in *Staphylococcus aureus* VraR activation. Proc Natl Acad Sci U S A 110:8525–8530. doi:10.1073/pnas.1302819110. 23650349 PMC3666669

[26] Doi A, Nakamura H, Shiro Y, Sugimoto H 2015 Structure of the response regulator ChrA in the haem-sensing two-component system of *Corynebacterium diphtheriae*. Acta Crystallogr F Struct Biol Commun 71:966–971. doi:10.1107/S2053230X15009838. 26249683 PMC4528925

[27] Baikalov I, Schröder I, Kaczor-Grzeskowiak M, Grzeskowiak K, Gunsalus RP, Dickerson RE 1996 Structure of the Escherichia coli response regulator NarL. Biochemistry 35:11053–11061. doi:10.1021/bi960919o. 8780507

[28] Holm L, Rosenström P 2010 Dali server: conservation mapping in 3D. Nucleic Acids Res 38:W545–W549. doi:10.1093/nar/gkq366. 20457744 PMC2896194

[29] Mariscotti JF, García-del Portillo F 2009 Genome expression analyses revealing the modulation of the Salmonella Rcs regulon by the attenuator IgaA. J Bacteriol 191:1855–1867. doi:10.1128/JB.01604-08. 19124574 PMC2648367

[30] Chakraborty S, Winardhi RS, Morgan LK, Yan J, Kenney LJ 2017 Non-canonical activation of OmpR drives acid and osmotic stress responses in single bacterial cells. Nat Commun 8:1587. doi:10.1038/s41467-017-02030-0. 29138484 PMC5686162

