# Supplementary figures and images for: Structural Basis for DNA Recognition by the Two-Component Response Regulator RcsB
Source: mBio. 2018 Feb 27;9(1):e01993-17. doi: 10.1128/mBio.01993-17 (PMC5829831; doi:10.1128/mBio.01993-17)

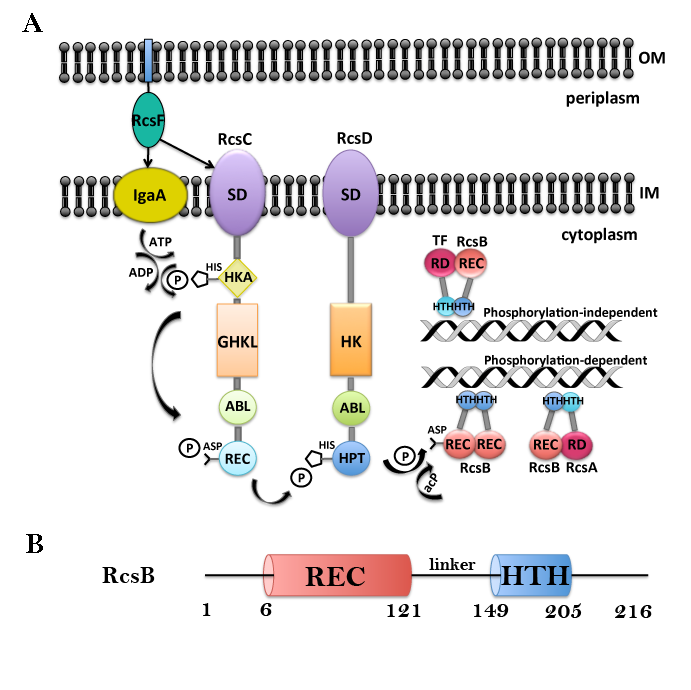

Supplement: FIG S1 [file mbo001183739sf1.tif]

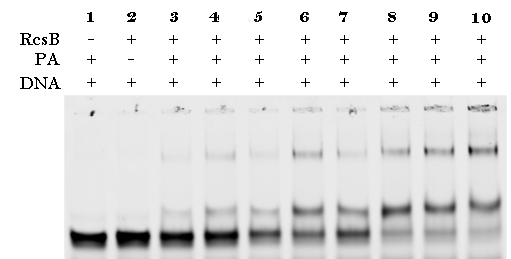

Supplement: FIG S2 [file mbo001183739sf2.tif]

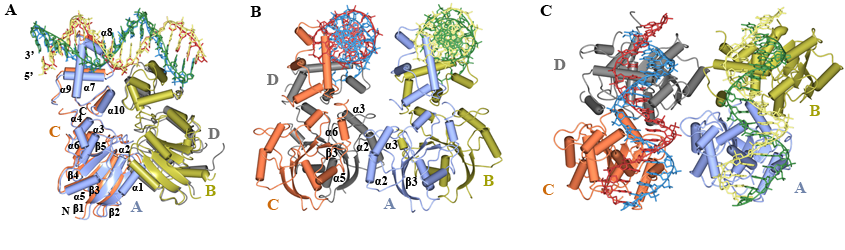

Supplement: FIG S3 [file mbo001183739sf3.tif]

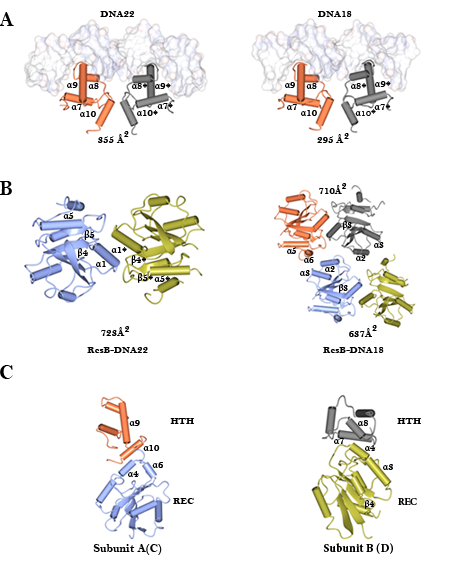

Supplement: FIG S4 [file mbo001183739sf4.tif]
