# Supplementary material for: Structural Basis for DNA Recognition by the Two-Component Response Regulator RcsB
Source: mBio. 2018 Feb 27;9(1):e01993-17. doi: 10.1128/mBio.01993-17 (PMC5829831; doi:10.1128/mBio.01993-17)
Supplement: TEXT S1 [file mbo001183739s1.docx]

**TEXT S1. Materials and Methods.**

**Design of DNA oligos.** DNA sequences used in our experiments presented below. Synthetic oligonucleotides of 22 or 18 base pairs were commercially synthesized by IDT Inc. in a single-strand form as 5’ end–labeled with IRDye 700, unlabeled or mutated. Sense strand and its complement of each pair were annealed according to the General Methodology for Infrared Electrophoretic Mobility Shift Assay (LI-COR Biosciences).

Strand Sequence

DNA fragment 22 base pair 5’-GATTTAGGAAAAATCTTAGATA

| Antisense DNA fragment 22 base pair 5’-TATCTAAGATTTTTCCTAAATC  Mutated DNA fragment 22 base pair 5’-GATTTAGAAAAAATTTTAGATA  Antisense mutated DNA fragment 22 base pair 5’-TATCTAAAATTTTTTCTAAATC  DNA fragment 18 base pair 5’-TTTAGGAAAAATCTTAGA  Antisense DNA fragment 18 base pair 5’-TCTAAGATTTTTCCTAAA  Mutated DNA fragment 18 base pair 5’-TTTAGAAAAAATTTTAGA  Antisense mutated DNA fragment 18 base pair 5’- TCTAAAATTTTTTCTAAA |
| --- |

**Recombinant protein production.** The DNA fragment encoding full-length RcsB from *E. coli* str. K12 substr. MG1655 (NCBI accession code AAC75277, GI: 1788546) was cloned into the pCA24n vector (chloramphenicol resistant) from the ASKA library with an additional 6-polyHis motif at the N-terminus for protein production. The clone was transformed into *E. coli* BL21(DE3)-magic competent cells (kanamycin resistant). The transformant was grown, expressed and purified, as described previously (1). The purity of protein was examined by SDS-PAGE gel. The protein sample solution contained 10 mM Tris-HCl pH 8.3, 500 mM NaCl and 5 mM β-mercaptoethanol. The DNA consensus sequences DNA22 and DNA18 and their complementary strands used for crystallization and *in vitro* studies are described above. DNA strands were annealed using a standard protocol described on the www.sigmaaldrich.com website. The DNA annealing buffer contained 10 mM Tris-HCl pH 7.5, 50 mM NaCl and 1 mM ethylenediaminetetraacetic acid.

**Crystallization and data collection.** Crystallization conditions were set up using the sitting-drop vapor-diffusion method at room temperature. The initial crystallization screening was set up using Corning 96-well plates and crystallization crystal screen Classics II from Qiagen Inc. 1 μL of screen solution was mixed with 1 μL of protein-DNA solution and equilibrated against 100 μL of reservoir screen solution. Prior to crystallization, the protein sample solution (defined above) at concentration 6 mg/mL was mixed with 1.5 mg/mL DNA 22 or DNA18, and incubated at room temperature for 30 min. Crystallization plates were incubated at 19^°^C. The initial crystallization screening conditions for crystals of RcsB-DNA22 complex were further optimized. Diffraction-quality crystals of RcsB-DNA22 complex were found in the drop holding 150 mM ammonium acetate, 0.1 M Tris-HCl pH 8.5, and 20% PEG3350. Crystals of RcsB-DNA18 complex were obtained from the drop with screen solution containing 200 mM NaCl, 0.1 M HEPES pH 7.5 and 25% PEG3350. Before data collection, crystals were transferred into their reservoir solutions and flash-frozen in LN_2_. Diffraction data were collected for RcsB-DNA22 and RcsB-DNA18 crystals at the Advance Photon Source at Argonne National Laboratory (Argonne, IL) at the LS-CAT beamlines at 100 K. The collected data for RcsB-DNA22 and RcsB-DNA18 were processed with the *HKL-2000* (2) *and XDS* (3)*,* respectively. X-ray data collection statistics are characterized in Table S1 in the supplemental material.

**Structure determination and refinement.** The co-crystal structures of RcsB-DNA22 and RcsB-DNA18 were determined by the molecular replacement method using *Phaser* (4). The structure of the putative RcsB receiver domain (PDB ID 5I4C) and the structure of the transcriptional regulator NarL HTH domain with DNA (PDB ID 1JE8) were used as the starting models. The structures were refined with *Phenix* (5) or *Refmac* (6) and manually rebuilt with *Coot* (7). The structures were validated using *MolProbity* (8). Structure determination and refinement statistics are summarized in Table S1 in the supplemental material. Structure figures were created in *CCP4mg* (9). The analysis of protein domain organization was performed on the Pfam database (http://pfam.xfam.org/).

**References**

1. **Hu LI, Chi BK, Kuhn ML, Filippova EV, Walker-Peddakotla AJ, Bäsell K, Becher D, Anderson WF, Antelmann H, Wolfe AJ. 2013.** Acetylation of the response regulator RcsB controls transcription from a small RNA promoter. J Bacteriol **195**:4174-4186.
2. **Otwinowski Z, Minor W.** 1997. **Processing of X-ray Diffraction Data Collected in Oscillation Mode.** Methods in Enzymolog **276**:Macromol Crystal part A:307-326, Carter CW Jr, Sweet RM, Eds, Academic Press, New York, NY.
3. **Kabsch W.** 2010. XDS*. Acta Crys* **D66**, 125-132.
4. **McCoy AJ, Grosse-Kunstleve RW, Adams PD, Winn MD, Storoni LC, Read RJ*.*** 2007. Phaser crystallographic software. J Appl Crystallogr **40**:658–674.
5. **Adams PD, Afonine PV, Bunkóczi G, Chen VB, Davis IW, Echols N, Headd JJ, Hung L-W, Kapral GJ, Grosse-Kunstleve RW, McCoy AJ, Moriarty NW, Oeffner R, Read RJ, Richardson DC, Richardson JS, Terwilliger TC, Zwart PH.** 2010. PHENIX: a comprehensive Python-based system for macromolecular structure solution. Acta Crys **D66**:213–221.
6. **Murshudov GN, Skubak P, Lebedev AA, Pannu NS, Roberto AS, Nicholls RA, Winn MD, Long F, Vagin AA.** 2011. REFMAC5 for the refinement of macromolecular crystal structures. Acta Crys **D67**:355–367.
7. **Emsley P, Cowtan K.** 2004. Coot: model-building tools for molecular graphics. Acta Crys **D60**:2126-2132.
8. **Chen VB, Arendall WB 3rd, Headd JJ, Keedy DA, Immormino RM, Kapral GJ, Murray LW, Richardson JS, Richardson DC.** 2010. MolProbity: all-atom structure validation for macromolecular crystallography. Acta Crys **D66**:12-21.
9. **McNicholas S, Potterton E, Wilson KS, Noble MEM.** 2011. Presenting your structures: the CCP4mg molecular-graphics software. Acta Crys **D67**:386-394.
