# Supplementary material for: Structural Basis for DNA Recognition by the Two-Component Response Regulator RcsB
Source: mBio. 2018 Feb 27;9(1):e01993-17. doi: 10.1128/mBio.01993-17 (PMC5829831; doi:10.1128/mBio.01993-17)
Supplement: TABLE S1 [file mbo001183739st1.docx]

| **Name** | **RcsB – DNA22** | **RcsB – DNA18** |
| --- | --- | --- |
| **Crystal parameters**  Resolution (last shell) (Å)  Space group  Unit cell parameters  a, b, c (Å)  α, β, γ (°)  Matthews coefficient (Å3/Da)  Solvent content (%) | 30.0-3.15 (3.15-3.2)  P6_1_  121.7, 121.7, 78.9  90, 90, 120  2.87  57.2 | 113.2-3.37 (3.37-3.39)  P2_1_  74.1, 113.2, 76.8  90, 115.4, 90  2.7  55.6 |
| **Data Collection**  Completeness (%)  No of unique reflections  I/σ(I)  R_merge_ (%)  CC_1/2_  Redundancy  Wilson B-factor (Å^2^) | 99.9 (100)  11611  15.2 (2.0)  0.09 (0.8)  0.7  4.9 (4.9)  100.9 | 93 (100)  15089  10.8 (2.1)  0.1 (0.6)  0.99  4.3 (4.5)  103 |
| **Refinement**  R (%)/R_free_ (%)  RMSD bond length (Å)  RMSD bond angle (°)  Average B value (Å^2^)  No of protein molecules in AU  No of residues  Protein  DNA | 18.3/25.1  0.015  2.02  121.9  2  386  44 | 19.8/26.8  0.003  0.5  90.4  4  792  72 |
| **Ramachandran analysis^#^**  Favoured (%)/n  Allowed (%)/n  Outlier (%)/n | 92.5/357  7.5/29  - | 96.2/743  3.8/29  - |

**TABLE S1** The crystal cell parameters, X-ray data collection, and structure refinement statistics.
